# Variation in SARS-CoV-2 Infection Risk and Socioeconomic Disadvantage Among a Mayan-Latinx Population in Oakland, California

**DOI:** 10.1001/jamanetworkopen.2021.10789

**Published:** 2021-05-21

**Authors:** Erin E. Esaryk, Paul Wesson, Jessica Fields, Francine Rios-Fetchko, Christina Lindan, Caryn Bern, Alicia Fernández

**Affiliations:** 1 Department of Epidemiology and Biostatistics, University of California, San Francisco; 2 University of California San Francisco Center for Vulnerable Populations at Zuckerberg San Francisco General Hospital, San Francisco; 3 University of California San Francisco Department of Medicine, Division of General Internal Medicine at Zuckerberg San Francisco General Hospital, San Francisco; 4 Latinx Center for Excellence, University of California, San Francisco

## Abstract

This cross-sectional study examines variation in SARS-CoV-2 infection risk and socioeconomic disadvantage among a Mayan-Latinx population in the Fruitvale, California, community.

## Introduction

US Latinx populations are disproportionally affected by the SARS-CoV-2 pandemic, with higher rates of infection and associated morbidity and mortality.^1^ Although often treated as homogeneous, members of Latinx communities vary by national origin, immigration status, and language.^2^ Oakland, California, is home to many Latinx individuals and an estimated 10 000 Mayan individuals, many of whom speak Indigenous languages.^3^ Early in the pandemic, community-based organizations (CBOs) in Oakland, California, observed a high frequency of infections among Latinx individuals in general and even higher frequency among Mayan individuals.^4^ Local CBOs, the University of California, San Francisco (UCSF), and public health authorities formed a collaborative to offer diagnostic testing in Fruitvale, a diverse neighborhood that has among the highest cumulative infection rates in Alameda County.^5^ Using data from the resulting SARS-CoV-2 testing event, we examined variation in infection risk and socioeconomic disadvantage within the Fruitvale community.

## Methods

This cross-sectional study was approved by the UCSF institutional review board and followed the Strengthening the Reporting of Observational Studies in Epidemiology (STROBE) reporting guideline. Free SARS-CoV-2 testing was provided for individuals of all ages on September 26 to 27, 2020. Adults gave verbal consent for themselves and for participating children. Anterior nasal swab samples were obtained for polymerase chain reaction (PCR) testing to detect the virus, and venous blood was collected to detect immunoglobin G antinucleocapsid antibodies. Adults completed a survey on sociodemographic characteristics at the testing event. Interviewers fluent in Spanish, 2 Mayan languages, and more than 4 other languages were available. We analyzed cross-sectional associations between demographic and socioeconomic indicators and SARS CoV-2 infection using χ^2^ tests and logistic regression analyses adjusted for age and sex and accounting for household clustering. Statistical significance was set at *P* < .05. Data analyses were conducted in Stata version 16 (StataCorp). Additional information regarding the methods appear in the eAppendix in the Supplement.

## Results

We tested 1186 individuals (1034 [87.2%] adults; 152 [12.8%] children; 610 [51.4%] female participants; mean [SD] age, 40.0 [18.3] years); 108 (9.1%) were Mayan individuals, 661 (55.7%) non-Mayan Latinx individuals, and 417 (35.2%) non-Latinx individuals. Compared with other Latinx individuals, Mayan individuals were more likely to live in households with 5 or more people (49 [53.3%] vs 152 [32.6%]; *P* < .005), report food insecurity (53 [62.4%] vs 172 [41.8%]; *P* = .001), have difficulty finding work due to the pandemic (12 [13.0%] vs 31 [5.4%]; *P* = .01), lack a regular medical practitioner (55 [64.7%] vs 340 [76.2%]; *P* = .03), and have no health insurance (35 [38.0%] vs 118 [20.7%]; *P* < .001) (Table 1). Mayan individuals were also more likely to have limited English proficiency compared with other Latinx participants (49 [58%] vs 187 [46%]; *P* = .04); 41 (44.6%) spoke a Mayan language at home. Mayan and non-Mayan Latinx participants had significantly greater odds of having a positive PCR test compared with non-Latinx participants (Mayan: adjusted odds ratio [aOR], 16.66; 95% CI, 3.54-78.41; *P* < .001; non-Mayan Latinx: aOR, 8.48; 95% CI, 1.91-37.67; *P* = .004). Mayan individuals were significantly more likely to have positive serology results compared with non-Latinx participants (aOR, 5.58; 95% CI, 2.13-14.65; *P* < .001) (Table 2).

**Table 1.** Demographic and Socioeconomic Characteristics of Study Population by Ethnicity

Characteristic	No. (%)			*P* value for comparison^a^		
	Non-Latinx^b^	Non-Mayan Latinx	Mayan	Non-Latinx vs Non-Mayan Latinx	Non-Mayan Latinx vs Mayan	Non-Latinx vs Mayan
**All participants**						
No.	417	661	108	NA	NA	NA
Age, y						
0-17	45 (10.8)	91 (13.8)	16 (14.8)	.19	.16	.07
18-49	223 (53.7)	358 (54.2)	68 (63.0)			
50-64	111 (26.8)	145 (21.9)	19 (17.6)			
≥65	36 (8.7)	67 (10.1)	5 (4.6)			
Sex^c^						
Female	203 (52.5)	356 (56.3)	51 (49.5)	.23	.20	.60
Male	184 (47.6)	276 (43.7)	52 (50.5)			
**Participants 18 y or older**						
No.	372	570	92	NA	NA	NA
Live in Fruitvale	143 (38.7)	275 (48.3)	66 (71.7)	.004	<.001	<.001
Work in Fruitvale	51 (13.7)	74 (13.0)	15 (16.3)	.72	.39	.54
Individuals in household, No.^d^				<.001		
1-4	247 (85.2)	314 (67.4)	43 (46.7)	<.001	<.001	<.001
≥5	43 (14.8)	152 (32.6)	49 (53.3)			
Income reduced due to pandemic	59 (15.9)	140 (24.6)	29 (31.5)	.002	.16	.001
Lost job due to pandemic	34 (9.1)	84 (14.7)	17 (18.5)	.01	.35	.01
Difficulty finding work due to pandemic	25 (6.7)	31 (5.4)	12 (13.0)	.40	.01	.05
No health insurance	29 (7.8)	118 (20.7)	35 (38.0)	<.001	<.001	<.001
Have regular health care practitioner^e^	219 (80.2)	340 (76.2)	55 (64.7)	.21	.03	.003
Household income <$50 000/y^f^	130 (58.8)	293 (77.1)	61 (87.1)	<.001	.06	<.001
Worried about ability to buy enough food^g^	99 (37.1)	207 (49.4)	52 (61.2)	.002	.05	<.001
Not enough food to last until paycheck^h^	91 (35.1)	172 (41.8)	53 (62.4)	.09	.001	<.001
Low English proficiency^i^	12 (18.8)	187 (46.2)	49 (58.3)	<.001	.04	<.001

Abbreviation: NA, not applicable.

^a^ *P* values by Mantel-Haenszel χ^2^ test.

^b^ Non-Latinx included participants who indicated the following racial identity categories: White (114 [27.5%]), Black (125 [30.1%]), Asian (65 [15.7%]), multiracial (22 [5.3%]), other (25 [6.0%]), NA (26 [6.3%]), and unknown (38 [9.2%]).

^c^ Data missing for 30 non-Latinx, 29 non-Mayan Latinx, and 5 Mayan participants.

^d^ Data missing for 82 non-Latinx and 104 non-Mayan Latinx participants.

^e^ Data missing for 99 non-Latinx, 124 non-Mayan Latinx, and 7 Mayan participants.

^f^ Data missing for 151 non-Latinx, 190 non-Mayan Latinx, and 22 Mayan participants.

^g^ Data missing for 105 non-Latinx, 151 non-Mayan Latinx, and 7 Mayan participants.

^h^ Data missing for 113 non-Latinx, 158 non-Mayan Latinx, and 7 Mayan participants.

^i^ Low proficiency corresponded to reporting speaking a language other than English at home and having answered not well or not at all to the question, “How well do you speak English?” Data on English proficiency collected only for participants who reported speaking a language other than English at home (66 non-Latinx, 423 non-Mayan Latinx, and 90 Mayan participants). Of those who reported speaking a language other than English at home, data on English language proficiency was missing for 18 non-Mayan Latinx and 6 Mayan individuals. An additional 97 Latinx and 78 non-Latinx individuals did not report the language spoken at home so were not asked about English proficiency.

**Table 2.** Association of Sex, Age, and Ethnicity With Positive SARS-CoV-2 PCR and Serology Test Results Among Participants in the Fruitvale Testing Event, September 2020

Characteristic	PCR test						Serology test					
	Tested, No.	Positive, No. (%)	OR (95% CI)	*P* value	aOR (95% CI)^a^	*P* value	Tested, No.	Positive, No. (%)	OR (95% CI)	*P* value	aOR (95% CI)^a^	*P* value
Sex^b^												
Female	570	14 (2.5)	1 [Reference]	NA	1 [Reference]	NA	454	48 (10.6)	1 [Reference]	NA	1 [Reference]	NA
Male	485	25 (5.2)	2.16 (1.12-4.17)	.02	2.25 (1.16-4.39)	.02	364	29 (8.0)	0.73 (0.48-1.11)	.15	0.70 (0.45-1.09)	.11
Age, y												
0-17	146	10 (6.9)	1 [Reference]	NA	1 [Reference]	NA	57	7 (12.3)	1 [Reference]	NA	1 [Reference]	NA
18-49	605	25 (4.1)	0.59 (0.28-1.25)	.17	0.57 (0.27-1.21)	.14	516	57 (11.1)	0.89 (0.36-2.19)	.80	1.06 (0.39-2.90)	.90
50-64	262	3 (1.2)	0.16 (0.03-0.83)	.03	0.16 (0.03-0.80)	.03	203	14 (6.9)	0.53 (0.19-1.46)	.22	0.61 (0.19-2.04)	.43
≥65	98	1 (1.0)	0.14 (0.02-1.15)	.07	0.15 (0.02-1.29)	.08	90	8 (8.9)	0.70 (0.20-2.41)	.57	0.77 (0.18-3.18)	.71
Ethnicity												
Non-Latinx	381^c^	2 (0.5)	1 [Reference]	NA	1 [Reference]	NA	304^d^	18 (5.9)	1 [Reference]	NA	1 [Reference]	NA
Non-Mayan Latinx	626	28 (4.5)	8.87 (2.00-39.43)	.004	8.48 (1.91-37.67)	.005	485	49 (10.1)	1.79 (0.86-3.69)	.12	1.76 (0.78-3.95)	.17
Mayan	102	9 (8.8)	18.34 (3.85-87.25)	<.001	16.66 (3.54-78.41)	<.001	76	19 (25.0)	5.30 (2.30-12.19)	<.001	5.58 (2.13-14.65)	<.001

Abbreviations: aOR, adjusted odds ratio; NA, not applicable; OR, odds ratio; PCR, polymerase chain reaction.

^a^ Models adjusted for sex and age. All models clustered on the household level to account for within-household correlation and correct standard errors.

^b^ Nonbinary or sex data missing from 5 participants.

^c^ Non-Latinx participants who received PCR testing included participants who indicated the following racial identity categories: White (107 [28.1%]), Black (114 [29.9%]), Asian (61 [16.0%]), multiracial (21 [5.5%]), other (23 [6.0%]), NA (21 [5.5%]), and unknown (34 [8.9%]).

^d^ Non-Latinx participants who received serology testing included participants who indicated the following racial identity categories: White (89 [29.3%]), Black (80 [26.3%]), Asian (54 [17.8%]), multiracial (18 [5.9%]), other (19 [6.3%]), NA (25 [8.2%]), and unknown (19 [6.3%]).

## Discussion

We found that Latinx participants were more likely to have current SARS-CoV-2 infection than non-Latinx participants, reflecting state and national trends.^1^ Our data highlight heterogeneity within the Latinx community, with Mayan individuals having even higher risk than other Latinx individuals. Findings related to socioeconomic disadvantage, including large household size, low income, and food insecurity, likely reflect the heightened susceptibility of Mayan individuals to the pandemic.^6^ In addition, limited English proficiency and access to health care pose challenges for effective public health messaging.

Limitations that reduce generalizability include that the study analyzed a convenience sample of those seeking testing, and that testing and medical referrals were prioritized over questionnaire completion, resulting in missing data. Unmeasured confounding factors could attenuate results.

This study underscores the need to consider heterogeneity within Latinx communities and to prioritize subgroups with higher risks, such as Mayan individuals, in health policies and outreach. Limited Spanish and English proficiency reduce this population’s access to health information and care. Few CBOs or public health departments have Mayan language speakers to provide information and perform contact tracing. Those that do are underresourced. Understanding differential risks within the heterogenous Latinx population can guide more efficient targeting of services. Failure to engage with communities with higher risks increases the likelihood of ongoing transmission and may hinder SARS CoV-2 vaccine uptake.

## Notes

Supplement. **eAppendix.** Supplementary Methods
