# Supplementary material for: Variation in SARS-CoV-2 Infection Risk and Socioeconomic Disadvantage Among a Mayan-Latinx Population in Oakland, California
Source: JAMA Netw Open. 2021 May 21;4(5):e2110789. doi: 10.1001/jamanetworkopen.2021.10789 (PMC8140375; doi:10.1001/jamanetworkopen.2021.10789)
Supplement: Supplement. — eAppendix. Supplementary Methods [file jamanetwopen-e2110789-s001.pdf]

Supplemental Online Content

Esaryk EE, Wesson P, Fields J, et al. Variation in SARS-CoV-2 infection risk and socioeconomic disadvantage among a Mayan-Latinx population in Oakland, California. *JAMA Netw Open*. 2021;4(5):e2110789. doi:10.1001/jamanetworkopen.2021.10789

eAppendix. Supplementary Methods

This supplemental material has been provided by the authors to give readers additional information about their work.

© 2021 Esaryk EE et al. *JAMA Network Open.*

eAppendix. Supplementary Methods

The SARS CoV-2 testing event in Fruitvale was organized by the Unity Council, a nonprofit community development organization; La Clínica de la Raza, a well-established network of Federally Qualified Health Centers; La Familia Counseling; the Resilient Fruitvale Taskforce, an umbrella group including Street Level Health Clinic, Native American Health Center and community advocates; Alameda County Public Health Department; and UCSF faculty and staff.

Recruitment.

Fruitvale community members were made aware of the testing opportunity via flyers that were distributed to neighborhood homes and at central locations (e.g. grocery stores); social media and local news sources (print, TV, and radio); and targeted outreach through local schools. Outreach was conducted in English, Spanish, Mam, and Arabic.

Data collection.

Participants could walk up or drive through to obtain SARS-CoV-2 testing and complete the survey either on foot or in their car. The survey was administered by trained volunteers using electronic tablets or by recording results onto paper-based questionnaires. The survey was written in English and Spanish, with in-person interpretation available in Mam, K'iche', Vietnamese, Arabic, Cantonese, Mandarin, and French.

Minors could participate only with verbal consent from at least one parent and completed an abbreviated survey. Participants could choose to provide an anterior-nares swab specimen for viral detection, and/or undergo a venous blood draw for antibody testing. All samples (nasal and blood) were collected by trained phlebotomists. Eluents from nasal swab samples were tested at the Chan-Zuckerberg BioHub using reverse transcription polymerase chain reaction (RT-PCR)

© 2021 Esaryk EE et al. *JAMA Network Open.*

assay for detection of the N and E genes of SARS-CoV-2. Serum samples were tested at the UCSF Clinical Microbiology Laboratory using the Abbot Architect reagents and platform.

Following testing, with or without survey completion, each adult participant received a bag with cleaning supplies, children's books and school supplies (if applicable), and a $20 Visa gift card as reimbursement for their time and effort. Clinical volunteers (RNs, MDs and medical students) disclosed positive PCR results by phone within 72 hours of testing, counseled participants on the meaning of the results and the need to isolate, offered to connect participants with a "Community Wellness Team" for additional support (e.g. food, cleaning supplies, assistance navigating financial assistance programs or relocating to an isolation and quarantine hotel if needed, etc.) during their isolation period. Positive antibody results and all negative PCR and serology results were disclosed via a secure online portal and text message. At the time of testing, participants were also provided with a phone number to call to receive all test results (as not all participants had cell phones or internet access). All test results and contact information were sent to the Alameda County Public Health Department for case investigation and contact tracing.

Analysis.

The study sample included all testing event participants who received either test. Pearson's Chi-squared tests were used to assess associations between sociodemographic variables and positive results by PCR and serology. Analyses were stratified by Latinx, non-Latinx, and Mayan ethnicity. We first performed bivariate logistic regression analyses to obtain odds ratios (OR) and 95% confidence intervals (CI), followed by multivariable logistic regression modeling, controlling for age and gender, to obtain adjusted ORs and 95% CI. Logistic regression models used the clustered sandwich estimator (vce(cluster clustvar) in STATA version 16) to account for intra-household correlation and ensure robust standard errors.

© 2021 Esaryk EE et al. *JAMA Network Open.*
